# Photoactive Nanomaterials

**DOI:** 10.3390/nano11010077

**Published:** 2021-01-01

**Authors:** Nurxat Nuraje

**Affiliations:** Department of Chemical and Materials Engineering, School of Engineering and Digital Science, Nazarbayev University, 53 Kabanbay Batyr Avenue, Nur-Sultan 010000, Kazakhstan; nurxat.nuraje@nu.edu.kz

With the depletion of carbon-based energy resources and the consideration of global warming, renewable energy is considered a promising energy source for future energy. Solar energy has shown the greatest potential to meet global energy needs with its ample source and free availability. Although primitive photovoltaic work gets credited to the silicon-based works done in the bell lab, recently, progress in the photovoltaic cell is significant. For example, many different types of solar cells have appeared, which include dye-sensitized solar cells, quantum-based solar cells, polymer solar cells, perovskite solar cells, etc. Here, we can point out the perovskite solar cell which reaches 25% efficiency within a short period. According to the literature search done on the Web of science, the number of publications on perovskite solar cells has increased exponentially within the last 10 years.

In addition to photovoltaic application, many research areas in utilization of solar energy have been extensively exploited to form distinct disciplines. One of them, photocatalytic water splitting, has molded a promising research pathway with exponentially increasing publications. Other fields include fuel generation from carbon dioxide, nitrogen conversion, photodegradation of organic and inorganic pollutants, and optoelectronic devices. Besides the fields mentioned, photoactive nanomaterials have been used in other fields. Experimental and theoretical investigations of solar energy conversion using various simulation software, from material design to reaction kinetics, lay the groundwork to improve the efficiency of the above applications. Furthermore, the fundamental study of photoactive nanomaterials in solar energy conversion, which is done through designing advanced functional materials and advanced experimental techniques, focuses on light harnessing, charge separation/recombination, and catalytic reaction kinetics.

Therefore, it is very important to deliver the recent research progress in the field of photoactive nanomaterials to the scientific community. Thus, the scope of this Special Issue not only covers a basic study of the solar energy conversion process with simulations but also targets certain applications including environmental remediation, optoelectronic devices, and solar fuel generation. This Special Issue includes six research articles and three review articles to focus on photoactive nanomaterials from fundamental research on their applications. Three research works [1,2,3] reported recent emerging materials, including 2D Bi_2_Se_3_ in optoelectronic applications, and discussed fundamental behaviors of dyes in confined media. Another research work reported by Maxim et al. [4] explores the enhancement of light harnessing in perovskite solar cells with embedded carbon quantum dots. Most of the articles are focused on photocatalytic solar fuel generation. Hydrogen generation kinetics is studied via silicon nanocrystals in aqueous media [5]. Nanocomposite-based photocatalysts including TiO_2_, SrTiO_3_, and Polyaniline (PANI) are also reviewed to give quick updates regarding photocatalytic water splitting [6,7,8]. Shabdan et al. updated with recent progress in a promising oxygen evolution of WO_3_-based photo-catalysts [9].

Therefore, I think it is essential to present a Special Issue which provide recent research works on photoactive nanomaterials, from fundamental studies to recent applications including optoelectronic devices, perovskite solar cells, and photocatalytic water splitting.

## References

[1] Sola-Llano R., Oliden-Sánchez A., Alfayate A., Gómez-Hortigüela L., Pérez-Pariente J., Arbeloa T., Hofkens J., Fron E., Martínez-Martínez V. (2020). White Light Emission by Simultaneous One Pot Encapsulation of Dyes into One-Dimensional Channelled Aluminophosphate. Nanomaterials.

[2] Wang S., Li Y., Ng A., Hu Q., Zhou Q., Li X., Liu H. (2020). 2D Bi_2_Se_3_ van der Waals Epitaxy on Mica for Optoelectronics Applications. Nanomaterials.

[3] Zeng Q., Guo S., Sun Y., Li Z., Feng W. (2020). Protonation-Induced Enhanced Optical-Light Photochromic Properties of an Inorganic-Organic Phosphomolybdic Acid/Polyaniline Hybrid Thin Film. Nanomaterials.

[4] Maxim A.A., Sadyk S.N., Aidarkhanov D., Surya C., Ng A., Hwang Y.-H., Atabaev T.S., Jumabekov A.N. (2020). PMMA Thin Film with Embedded Carbon Quantum Dots for Post-Fabrication Improvement of Light Harvesting in Perovskite Solar Cells. Nanomaterials.

[5] Mussabek G., Alekseev S.A., Manilov A.I., Tutashkonko S., Nychyporuk T., Shabdan Y., Amirkhanova G., Litvinenko S.V., Skryshevsky V.A., Lysenko V. (2020). Kinetics of Hydrogen Generation from Oxidation of Hydrogenated Silicon Nanocrystals in Aqueous Solutions. Nanomaterials.

[6] Fu Y.-S., Li J., Li J. (2019). Metal/Semiconductor Nanocomposites for Photocatalysis: Fundamentals, Structures, Applications and Properties. Nanomaterials.

[7] Bakbolat B., Daulbayev C., Sultanov F., Beissenov R., Umirzakov A., Mereke A., Bekbaev A., Chuprakov I. (2020). Recent Developments of TiO_2_-Based Photocatalysis in the Hydrogen Evolution and Photodegradation: A Review. Nanomaterials.

[8] Sultanov F., Daulbayev C., Azat S., Kuterbekov K., Bekmyrza K., Bakbolat B., Bigaj M., Mansurov Z. (2020). Influence of Metal Oxide Particles on Bandgap of 1D Photocatalysts Based on SrTiO_3_/PAN Fibers. Nanomaterials.

[9] Shabdan Y., Markhabayeva A., Bakranov N., Nuraje N. (2020). Photoactive Tungsten-Oxide Nanomaterials for Water-Splitting. Nanomaterials.

